# Bringing oral healthcare to where people are: an implementation evaluation of an oral health education program on an inpatient mental health unit

**DOI:** 10.3389/froh.2026.1986995

**Published:** 2026-10-30

**Authors:** Nesya Walls, Luke Di Paolo, Jennifer Ridsdale, Rio Anzolin, Grace Park, Elana Commisso, Margo Reilly

**Affiliations:** 1 Windsor Family Health Team, Windsor, ON, Canada; 2 Department of Mental Health, Windsor Regional Hospital, Windsor, ON, Canada; 3 Corporate Office of Research, Windsor Regional Hospital, Windsor, ON, Canada; 4 Executive Committee, Windsor Regional Hospital, Windsor, ON, Canada

**Keywords:** health equity, implementation science, integrated care, mental health, oral health, oral health promotion, people-centred care, team-based care

## Abstract

**Background:**

In Canada, where access to healthcare does not ordinarily depend on ability to pay, oral healthcare has been the persistent exception. People living with serious mental illness are among those least likely to access oral healthcare. Oral health services have been integrated into existing healthcare settings, mostly as clinical treatment; however, the upstream work of education, prevention and promotion is less well documented. The Windsor Family Health Team's Oral Health Education Program (OHEP) brings upstream oral health services to people where they already receive care. Developed for primary care, it was subsequently delivered by a registered dental hygienist on an inpatient mental health unit at Windsor Regional Hospital.

**Methods:**

We conducted a multi-source implementation evaluation, drawing on the RE-AIM framework, Proctor's implementation outcomes and the updated Consolidated Framework for Implementation Research to examine reach, adoption, acceptability, appropriateness and feasibility. Data came from patient questionnaires, a unit staff survey, and an interview with the program's Oral Health Educator. The study was originally designed as a prospective cohort evaluation, but too few participants completed follow-up questionnaires to support conclusions about change over time.

**Results:**

Eighty-seven patients enrolled and three withdrew, leaving 84; 35 responded at 7 days, 38 at 30 days and 15 at 90 days. Participants included people adversely impacted by determinants of health: 9.5% identified as Indigenous, 52.4% reported an income below CAD $20,000, and 29.8% had been unhoused in the past year. Of 61unit staff surveyed, 88.5% had referred a patient to the program, 88.5% considered it well or very well integrated into the unit workflow, and 93.4% were satisfied or very satisfied with its implementation. Almost all responding patients said they would recommend it. The program was delivered two half-days each week, which limited how many patients could be seen.

**Conclusion:**

Upstream oral health services were integrated into this inpatient mental health setting and reached people who face barriers to accessing oral healthcare. Study participant attrition is itself a finding, and points to compounding gaps in policy, practice, and what counts as evidence. This is an equity problem as much as a methodological one.

## 1 Introduction

In Canada, where access to healthcare does not ordinarily depend on the ability to pay, oral healthcare—from education, prevention and promotion to dental treatment—has been the persistent exception: access has depended largely on private insurance, employer benefits, out-of-pocket payment, or enrolment in limited public coverage programs for specified groups (1–3). The many Canadians who cannot pay, and who face barriers to accessing existing funding supports and healthcare more broadly, are at a higher risk of poorer oral health, and by extension, poorer overall health, quality of life and wellbeing outcomes. This paper reports the findings of an implementation evaluation of an oral health education program, developed in a primary care setting and then adapted and implemented on an inpatient mental health unit.

Over one in four Canadians report living with oral pain or avoiding food because of problems with their mouth (4), and roughly one in four people aged 12 and older avoids dental care because they cannot afford it (5). This burden is not borne equally. Gaps in oral health coverage and barriers to accessing oral healthcare disproportionately affect people living on low incomes, people experiencing serious mental illness, Indigenous peoples, newcomers, people without stable housing, and people ageing without benefits (6–9). This is a clear example of the inverse care law: those with the greatest need receive the least care (7, 10).

A growing body of evidence demonstrates the ways in which oral health conditions interact with and compound other health conditions across the life course: periodontal disease is associated with cardiovascular disease and diabetes; chronic oral infection contributes to systemic inflammation; poor oral health is implicated in respiratory illness and adverse pregnancy outcomes (6, 7). Individuals and groups who are least able to access timely care are at greater risk of experiencing the deleterious consequences of oral health conditions. This is particularly true for people living with serious mental illness and people adversely impacted by determinants of health (9, 11–13).

The link between mental illness and poorer oral health is well documented: the relationship between oral and mental health runs in both directions, yet oral health remains an often overlooked aspect of mental health care (14). People living with serious mental illness are 2.7 times more likely to lose all of their teeth than the general population (14). Psychiatric medications commonly cause xerostomia (dry mouth), which accelerates dental caries and periodontal disease and creates persistent discomfort that further compounds the burden of illness (15). Avolition, a diminished ability to initiate and sustain goal-directed activities, may interfere with oral self-care and help-seeking among people living with serious mental illness, while experiences of shame and stigma can create additional barriers to accessing dental care (14, 16, 17). Dental phobia is also more prevalent among people with mental health disorders, which makes the standard care pathway, a patient-initiated dental office appointment, unlikely to be used (18, 19). These risks are compounded by hospitalisation. Studies consistently demonstrate that oral health deteriorates during inpatient admission as a consequence of acute illness, medication use, and limited access to oral hygiene support (20, 21). Nor does discharge resolve the problem: inpatients with psychiatric and substance use disorders access dental services irregularly, and the barriers are not only financial (22).

The link between social determinants and oral health is equally well documented. People experiencing low income, housing instability, and food insecurity face higher rates of tooth loss, untreated decay, and oral pain, and are simultaneously the least likely to access preventive dental care (9, 12). In Canada, income-related inequalities in oral health are greater than income-related inequalities in general health indicators (9, 13, 23). These are not only risk factors for oral disease; they are also the reasons why the standard care pathway is not accessible for this population.

Whether care is inaccessible, delayed, or simply not sought, the need does not disappear. Instead, it resurfaces elsewhere in the healthcare system, often in settings that are neither designed nor funded to provide the necessary care, resulting in poorer population health outcomes and creating greater costs for the system. In the province of Ontario alone, nearly 60,000 emergency department visits for dental problems were recorded in 2017, at an estimated average cost of CAD $513 per visit and over CAD $31 million in total (24). Where a dental problem leads to a hospital admission the mean cost has been estimated at CAD $7,367 (25). Not only are these visits costly, they also do not address the underlying disease that prompted them, as patients are typically treated with medications for symptom management, while the condition itself goes untreated (26).

This situation is not unique to Canada. Oral diseases affect an estimated 3.5 billion people worldwide (6) and persist not because they are untreatable, but because current treatment-dominated approaches are not organised to address the upstream factors, and underlying causes of disease, or to reach those with the greatest need (7, 27). Addressing this requires more than expanding how dental treatment is paid for and by whom; it requires rethinking how and where oral healthcare is delivered. The principle that essential healthcare must be universally accessible, and delivered as close as possible to where people live and work, first articulated in the Declaration of Alma-Ata in 1978, provides the foundation for this reorientation (28).

Over the past decade, there has been growing recognition, both internationally and in the Canadian context, that oral health can no longer be treated as separate from overall health, and that longstanding access and equity gaps need to be addressed (6, 29, 30). Internationally, the World Health Organization's (WHO) Global Strategy and Action Plan on Oral Health 2023–2030 is the most prominent example of this recognition. The strategy positions oral health as a global public health priority and calls for a shift away from treatment-dominated models towards comprehensive care that spans prevention, promotion, and disease management, as well as the integration of oral health into primary healthcare systems and universal health coverage frameworks (29). This shift has also included growing recognition that oral and mental health are closely linked and should not be addressed in isolation (14). In the United States, the National Council for Mental Wellbeing has published a framework for coordinating and integrating oral health, mental health and substance use treatment services (31). In the Canadian context, this reorientation is beginning to take hold. The creation of a first-ever National Oral Health Research Strategy, launched in June 2024, which positions health equity and implementation science as research and policy priorities, is an encouraging example (32). More concretely, the introduction of the Canadian Dental Care Plan (CDCP), which began enrolling its first recipients in 2023 and continues to expand eligibility, represents the most significant federal investment in oral healthcare in a generation (33).

The CDCP is a meaningful and overdue step towards addressing the coverage gap that has persisted since Medicare was established. For people who experience barriers to accessing oral healthcare, however, the CDCP does not resolve the access challenge. Its eligibility conditions, including the annual filing of a tax return and the need to actively register for coverage (34), cannot be met by many of those with the greatest need: people experiencing homelessness or housing instability, those not attached to primary care, or navigating the functional disruptions that accompany serious mental illness, as well as many newcomers and individuals who face practical barriers to registration itself. For those who qualify and register, the CDCP covers dental treatment and preventive clinical services; however, it does not cover the full continuum of comprehensive oral health services, which begins with education and promotion, that address the upstream determinants driving oral health conditions (33). The oral health education program discussed in this paper provides education and oral health promotion services that the CDCP does not cover, at no cost and without requiring enrolment in the CDCP.

Approaches to mitigating these access barriers and improving oral health outcomes exist (35, 36), and most of these approaches are guided by a common principle: oral health services must be delivered in the settings where people already are, rather than requiring people to navigate a separate system. This principle is the foundation of the WHO's Framework on Integrated, People-Centred Health Services, which extends the universal-access commitment of Alma-Ata into a blueprint for how care should be designed, organised, and delivered (28, 37). Integration, the deliberate embedding of oral health education, prevention, and promotion into existing health and social care settings, is the approach the evidence supports (35, 36). In inpatient settings, where oral health services have been embedded in care pathways, the evidence shows reductions in health complications and improvements in patient-reported oral health (20). Evidence from community-based and outreach settings similarly demonstrates that bringing oral healthcare to where people are, rather than waiting for them to present to a dental office, improves both reach and outcomes among people who would not otherwise access care (38–40). Much of this evidence, however, comes from outreach models that function as supplementary access points rather than as integrated service delivery options (39, 40). Where oral healthcare has been embedded into existing settings, evidence of success has primarily focused on clinical treatment; the upstream work of education, prevention, and promotion is less well documented. The evidence on educational, prevention and promotion-focused interventions that does exist is promising: early intervention builds knowledge, self-efficacy, and connection to care, which improves oral health behaviours and reduces the progression of disease, particularly among populations with significant unmet need (41, 42). What remains far less understood is how these upstream services are best implemented, adapted, and delivered in different settings (27): whether they reach the populations the dental system does not, how staff adopt them, and how they can be evaluated.

The main barrier to integration is not a lack of evidence that it works; it is the difficulty of mobilising infrastructure, workforce, and funding. In an ideal system, the full continuum of oral healthcare would be integrated into multiple settings that people can access easily, or where they receive services over a prolonged period of time, including primary care, acute care, and community-based settings, with registered dental hygienists and dentists providing direct clinical services alongside other members of the care team. In the current funding and policy environment, that model is not available to most people who experience the greatest barriers to accessing oral healthcare.

The Oral Health Education Program (OHEP) was developed by the Windsor Family Health Team (WFHT), a community-governed comprehensive primary healthcare organisation, as an equity-oriented, population-health focused response to the disparities in oral health outcomes experienced by members of the communities it serves. The WFHT designed the program for adaptation and spread across diverse health and community settings where people who experience poorer oral health outcomes and barriers to accessing oral healthcare already receive services and supports; this design intent is set out in the program's concept paper, prepared by the WFHT. The WFHT first implemented the program in its own primary care setting; oral health education, prevention and promotion services were integrated into its existing service offerings, for individuals and groups it serves who cannot access the mainstream dental care system. The WFHT later spread the program into mobile outreach clinic settings, and the Windsor Regional Hospital's Executive Committee then approved its implementation on the hospital's inpatient mental health unit, where it was adapted for that setting. The unit is precisely the type of setting the program was designed for, because patients on the unit live with serious mental illness and are frequently also adversely impacted by determinants of health, which places them at higher risk of poor oral health.

This study was originally designed as a prospective cohort evaluation of whether the OHEP changed oral health knowledge, behaviours and quality of life of patients on the inpatient mental health unit. However, because most participants did not complete follow-up questionnaires after discharge, we amended the study design to support an implementation evaluation (see Section 2.1). We examined who the program reached, how it was delivered and adopted, and its acceptability and feasibility, from the perspectives of patients, unit staff who referred patients to the program, and the registered dental hygienist who delivered the program, the Oral Health Educator (OHE). We further consider what evaluating a program of this kind, with this population, revealed about how such research can be conducted and what types of evidence are meaningful to support further spread and scalability of upstream oral health services in diverse settings.

## 2 Materials and methods

### 2.1 Study design

The study was designed as a prospective cohort evaluation of the OHEP as delivered on the inpatient mental health unit at Windsor Regional Hospital. Patients were followed at four time points: baseline, 7 days, 30 days, and 90 days. Data were collected using structured questionnaires and the Oral Health Impact Profile-14 (OHIP-14), a validated patient-reported outcome tool (43). The study was non-interventional; the program was delivered as part of the standard of care.

The original prospective cohort study could not be completed as designed because of patient participant attrition. Recruitment ran from April 2024 to March 2025, and patient participants were lost to follow-up throughout; most patients did not complete the 90-day questionnaire (see Results). Patient data collection ended in May 2025, by which point we suspected that attrition resulted in too few follow-up responses to assess change over time. We nonetheless sent the patient data for statistical analysis in June 2025, to confirm whether change could be assessed and to see what else they could show. Delays in the research process, including a change of statistician, meant the analysis was not completed until November 2025. The analysis confirmed our suspicion that the program's effects could not be assessed. The data did show two things: who the program reached, and how patients rated it. We therefore decided to examine how the program had been implemented, and amended the study to collect further data from the unit staff and the OHE through a survey and an interview. We treat the difficulty of following this population in a research study as a finding in its own right.

### 2.2 Implementation evaluation and frameworks

An implementation evaluation asks how a program is put into practice in a particular setting: how it is adopted and adapted, and what helps or hinders its uptake (44). Whether a program is taken up depends largely on contextual factors, not only on how effective it is (45, 46). Accordingly, implementation outcomes differ from effectiveness outcomes: rather than measuring the program's effect, they capture whether it is accepted, adopted, appropriate and feasible where it is delivered, and how well it has been put into practice (47). Thus, in line with an implementation evaluation approach, we set out to examine who the program reached, whether staff on the unit adopted it, whether those involved found it acceptable and appropriate, whether it could be delivered in this setting, and what helped and hindered its delivery.

We used a multi-source implementation evaluation design, drawing on quantitative data from patient questionnaires and a staff survey, and qualitative data from an interview with the OHE. We analysed data from all three sources together, reporting findings by implementation outcomes alongside the determinants that shape implementation (see Section 2.5). Three implementation frameworks and one taxonomy of implementation outcomes guided the evaluation, each with a distinct purpose. We used the RE-AIM (Reach, Effectiveness, Adoption, Implementation, Maintenance) framework to define reach, adoption and implementation (48). We used Proctor's taxonomy of implementation outcomes to define acceptability, appropriateness and feasibility (47). We used the updated Consolidated Framework for Implementation Research (uCFIR) to identify the factors that helped or hindered the program's implementation on the unit (49). We used the Framework for Reporting Adaptations and Modifications-Expanded (FRAME) to develop the interview questions on how the program was adapted (50).

No single framework covered everything we needed. The uCFIR addresses the determinants of implementation but does not define outcomes (49); its developers draw on RE-AIM and Proctor's taxonomy for that purpose, because they cover different outcomes (51). We used RE-AIM, Proctor's taxonomy and the uCFIR to develop the staff survey questions and the interview guide. Supplementary Table S1 lists each measure with its data source, implementation outcome, framework and, where applicable, uCFIR determinant. For measures from the patient questionnaires and staff survey, quantitative results are provided; findings from the interview are reported in the Results section.

We measured reach among patients admitted to the unit and adoption among unit staff, and describe implementation in terms of how the program was delivered and adapted on the unit. We did not assess effectiveness, for the reasons stated in Section 2.1, or maintenance, the program's sustained delivery over time. Staff and educator perspectives on whether the program could be sustained and spread are reported with the determinants of implementation. We operationalised the uCFIR for this study by defining the OHEP as the innovation and the inpatient mental health unit as the inner setting, and drew on constructs from its innovation, inner setting and implementation process domains. Because the evaluation focused on delivery within a single organisation, we did not assess outer-setting determinants (49). We did not measure fidelity to program components; instead, we assessed fidelity in terms of the program's underlying principles and goals, as set out in the program's concept paper (52). We return to this in the Discussion.

### 2.3 Setting and program

The study was conducted on the inpatient mental health unit at Windsor Regional Hospital's Ouellette campus, in Windsor, Ontario, Canada. The unit comprises two physical spaces on one floor with 65 beds between them and is locked. Admissions to the unit are both voluntary and involuntary in a fluctuating ratio of roughly 70:30. During the study recruitment window, 1,226 unique patients were admitted to the unit. The average length of stay is approximately 12 days, and for many patients admitted to the unit, hospitalisation is the only stable contact with the health system over an extended period of time.

The OHEP was developed by the WFHT for primary care, and was designed to be adaptable to other settings. Its underlying principles are that services are provided at no cost to people without access to dental care, are integrated into the settings where people already receive care, and are tailored to the individual in line with a people-centred approach. It was brought to the unit in December 2022 with minor adaptations to accommodate an inpatient setting. The OHE, a registered dental hygienist employed by the WFHT, co-developed the program and has completed additional professional development in trauma-informed care. The program comprises a defined set of core components delivered one-on-one by the OHE at an individual patient's pace, and using a trauma-informed approach: individualised oral health education and self-care coaching; complete oral cancer screening, including the oropharyngeal area; caries risk assessment; preventive applications, including fluoride varnish, silver diamine fluoride, and desensitising treatments where indicated; symptom management, including relief of xerostomia; and the provision of oral hygiene supplies and educational materials. Each patient receives the components that are relevant to their specific oral health needs.

Patients reached the program by referral from unit staff, through the OHE approaching them directly, and by word of mouth from other patients. Taking part required capacity to consent, which was assessed by the patient's care team as part of routine clinical care. Patients who did not have capacity to consent at the time, including those for whom a substitute decision-maker was providing consent under Ontario's Mental Health Act (Form 33), and patients who were not well enough to take part were not seen by the OHE. Patients admitted to the unit involuntarily who had capacity to consent could access the program. Patients could stop an encounter at any time. At the end of an encounter patients received a personalised oral health plan, and where appropriate, referral information was provided at discharge. The OHE delivered the program on the unit two half-days each week. Since the program began being offered on the unit in December 2022, the OHE recorded 777 program encounters with patients. A patient may have had more than one encounter with the OHE, so this is not a count of unique patients; it reflects the uptake of the OHE's services on the unit.

### 2.4 Participants and data collection

The evaluation drew on three principal data sources: patient questionnaires (the study questionnaire and the OHIP-14); a staff survey; and an interview with the OHE. Clinical information, including the OHE's consultation notes, patient medication lists, and clinical conditions and diagnoses, was also abstracted from the patient chart at baseline, to support the original effectiveness design; because the study was reported as an implementation evaluation, these data were not used.

#### 2.4.1 Patient data

We recruited patients who had been admitted to the unit, aged 16 years or older, had completed the oral health education session within the preceding 24 h, and were proficient in written and spoken English. Verbal interpretation was available during a patient's stay on the unit, but not for follow-up after discharge; translation of study documents was not available at any point. English proficiency was therefore a condition of participation, as the consent process and follow-up questionnaires required that participants and researchers communicate directly. No patient was recorded as excluded on grounds of language, and the OHE reported that all patients they saw on the unit spoke English; we consider the implications of this requirement in the Limitations. Participation also required that patients provide a telephone number or email address at which they could be reached after discharge. The first participant was enrolled on April 23, 2024 and the last baseline questionnaire was administered on March 18, 2025.

Patients received the program first, and were then invited to take part in the research study. Taking part in the program required capacity to consent, which had been assessed by the patient's care team (Section 2.3). After the education session, the OHE introduced the study and told patients that taking part was voluntary; because they had already received the program, declining to participate in the study had no bearing on their ability to access it. Where a patient agreed to participate, the OHE notified a research team member, who confirmed eligibility, discussed the study with the patient and obtained written consent, either immediately or within 24 h where the patient wanted more time. Capacity to consent was not reassessed at enrolment, as the research team relied on the care team's assessment, made before the patient received the program (Section 2.3). The research team repeated that participation in the study was voluntary during the consent discussion and at each follow-up contact point and that participants could withdraw from the study at any time.

The baseline questionnaire captured study participants' dental history and access to oral healthcare, and their knowledge, attitudes and behaviours related to oral health, in addition to self-reported sociodemographic information. The follow-up questionnaire repeated these items and asked about patients' use of the program and any self-reported changes in their oral health. The OHIP-14 was administered alongside the study questionnaire at baseline, 30 days and 90 days, to assess the impact of oral health problems on patients' quality of life. Responses to all patient questionnaires were entered directly into REDCap, a secure web-based data capture platform (53). The baseline and follow-up questionnaires are provided in Supplementary Files S1, S2, respectively.

#### 2.4.2 Staff data

Staff working on the unit, excluding any who were involved in the program delivery or the research study, were invited to complete a survey about their perspectives on the program. Staff received a survey link by email from a unit administrator with no identified conflict of interest; the survey was open from February 9 to February 23, 2026. Both the invitation to complete the survey and the consent information at the start of the survey stated that responses were anonymous and that taking part in the survey was voluntary. The survey asked about staff members' awareness of the program, their experience of its implementation, and their view of its value; it took five to ten minutes to complete and was hosted on REDCap. The full staff survey is provided in Supplementary File S3. We developed the survey items for this study using RE-AIM, Proctor's taxonomy and the uCFIR (Section 2.2). Items on acceptability and appropriateness asked about this program on this unit rather than using generic validated measures, and most other items, such as awareness of the program's availability and referral, had no validated equivalent, to our knowledge. We consider this in greater detail in the Limitations.

#### 2.4.3 OHE data

The OHE, as the only person delivering the program, participated in a semi-structured interview on February 2, 2026, which asked about the program's design, its adaptation to the unit setting, and factors that shaped its delivery. The interview guide was structured in alignment with uCFIR constructs, Proctor's implementation outcomes, and adaptation-related constructs from the FRAME framework (50). The interview guide is provided in Supplementary File S4. NW, a WFHT colleague of the OHE in a non-supervisory role, conducted the interview by video call. The interview lasted 74 min. It was audio-recorded for transcription, and the transcript was returned to the OHE to confirm its accuracy; the recording was deleted once transcription was complete.

### 2.5 Data analysis

As described in Section 2.1, data from patient questionnaires and the OHIP-14 were analysed by statisticians, in IBM SPSS Statistics (version 31.0.1.0). For this evaluation the analysis was descriptive: categorical variables were described as percentages. We examined the sociodemographic profile of study participants, and report responses to individual follow-up items as counts of respondents at each time point. The statisticians also ran the analyses the original design required: continuous variables were tested for normality using the Shapiro–Wilk test and were non-normally distributed; the Wilcoxon signed-rank test was used to compare paired samples, and the Kruskal–Wallis and Mann–Whitney U tests to compare independent samples. Where a participant did not complete both items in an OHIP-14 domain, that domain was excluded from analysis. Too few patients completed follow-up questionnaires to support conclusions, so we do not report the results of these analyses.

Staff survey results were analysed descriptively, with responses summarised as counts and percentages. Because the survey was anonymous, individual responses could not be linked and inferential analyses were not possible. The interview with the OHE was transcribed and analysed deductively: EC coded the transcript against the frameworks described above following established approaches in qualitative coding methods (54, 55), and NW, who conducted the interview, independently reviewed the coding and the construct assignments. In the interview, the OHE rated eight potential determinants of implementation, such as support from staff and leadership, space and patient engagement. Each was rated twice on a scale of 1 to 5: once for how much it hindered delivery, and once for how much it helped. We mapped their ratings to the corresponding uCFIR determinants. Determinants rated 4 or 5 for hindering are reported as barriers, and those rated 1 or 2 as not barriers; enablers were drawn from the ratings for helping together with the OHE's account in the interview. Because the qualitative component was a single interview, we did not generate themes; interview findings are reported descriptively as they relate to each outcome and determinant.

We analysed findings from all data sources together and organised them by the implementation outcomes specified by RE-AIM and Proctor: reach, adoption, acceptability, appropriateness, and feasibility. EC mapped each survey item and interview finding to its implementation outcome and, where applicable, uCFIR determinant (Supplementary Table S1), and NW reviewed the mapping; discrepancies in the coding and the mapping were resolved by discussion. We drew on the uCFIR to identify the determinants that shaped the program's adoption and delivery; they are reported alongside outcomes and summarised at the end of the Results.

### 2.6 Researcher positionality

Two authors carried out the analysis. NW, who conducted the interview and reviewed the analysis, is employed by the WFHT, the organisation that developed and delivers the program, and co-authored its concept paper. NW has experience in qualitative data collection and works in quality improvement and decision support, which may incline NW towards practical, improvement-focused interpretations. As an employee of the WFHT, NW also has an interest in the program's success. In reviewing the analysis, NW therefore looked in particular for findings that did not fit what was expected. EC, who coded the interview and mapped the survey items and interview findings to the frameworks, is a consultant contracted by the WFHT for specific projects, including this manuscript, and has no role in the program. EC's background is in implementation and quality improvement work in primary care in Ontario, with a focus on building equity-oriented programs, which leads them to see implementation as dependent on context and relationships; this may have influenced how determinants were read. JR, the OHE and the only interview participant, co-developed the program and is a co-author of this paper; their account may reflect personal investment in it, which we take into account in interpreting the findings and in the Limitations. We took several steps to strengthen the trustworthiness of the analysis and mitigate confirmation bias: the coding and mapping were done by an author who had no role in the program and had not conducted the interview, and were reviewed by the author who had; JR checked the transcript for accuracy; and interview findings were read alongside the staff survey.

### 2.7 Ethics

The study received Category A, Full Approval from the Windsor Regional Hospital Research Ethics Board (REB# 24-481; March 20, 2024) and from the University of Windsor Research Ethics Board (REB# 24-156; October 28, 2024) and was conducted in accordance with the Tri-Council Policy Statement (TCPS2, 2022) and the Personal Health Information Protection Act (2004). Patients independently provided written informed consent; staff survey completion was taken as implied consent; and the OHE provided verbal consent, recorded at the time of interview. An amendment adding the staff survey and the OHE interview was approved in January 2026.

Study data were held under a participant study identifier, with a master linking log stored separately in a password-protected hospital network folder accessible only to the research team. Student research assistants accessed the study folder on site only; other members of the research team had remote access to the hospital network as part of their staff roles. Questionnaire data were held in a separate REDCap project from the signed consent forms: consent forms carried identifiable information, questionnaires did not, and questionnaires were organised by participant study identifier. Data held in REDCap were restricted to authorised users through multi-factor authentication. Findings are reported in aggregate, with any category of fewer than five participants combined to prevent re-identification. Study data will be retained for five years.

## 3 Results

Of 91 patients invited to participate in the study, 87 consented and enrolled (Figure 1). Three patients withdrew their consent and data from the study. Of the 84 patients remaining in the study, all of whom completed the baseline questionnaire, 35 patients responded to the 7-day follow-up questionnaire, 38 responded at 30 days and 15 responded at 90 days. Not every respondent answered every item in the questionnaires, so denominators vary across measures. Because the unit's mean length of stay was 12 days and the follow-up schedule extended to 90, study retention was low, and the effectiveness question the study set out to answer could not be answered. The OHIP-14 scores are therefore not reported. We do report what the data support: who the program reached, how staff experienced it and whether it was acceptable and feasible to deliver, as well as the factors that shaped its delivery, which we name where they arise and draw together at the end of the section. Table 1 summarises the main quantitative results.

**Figure 1:**
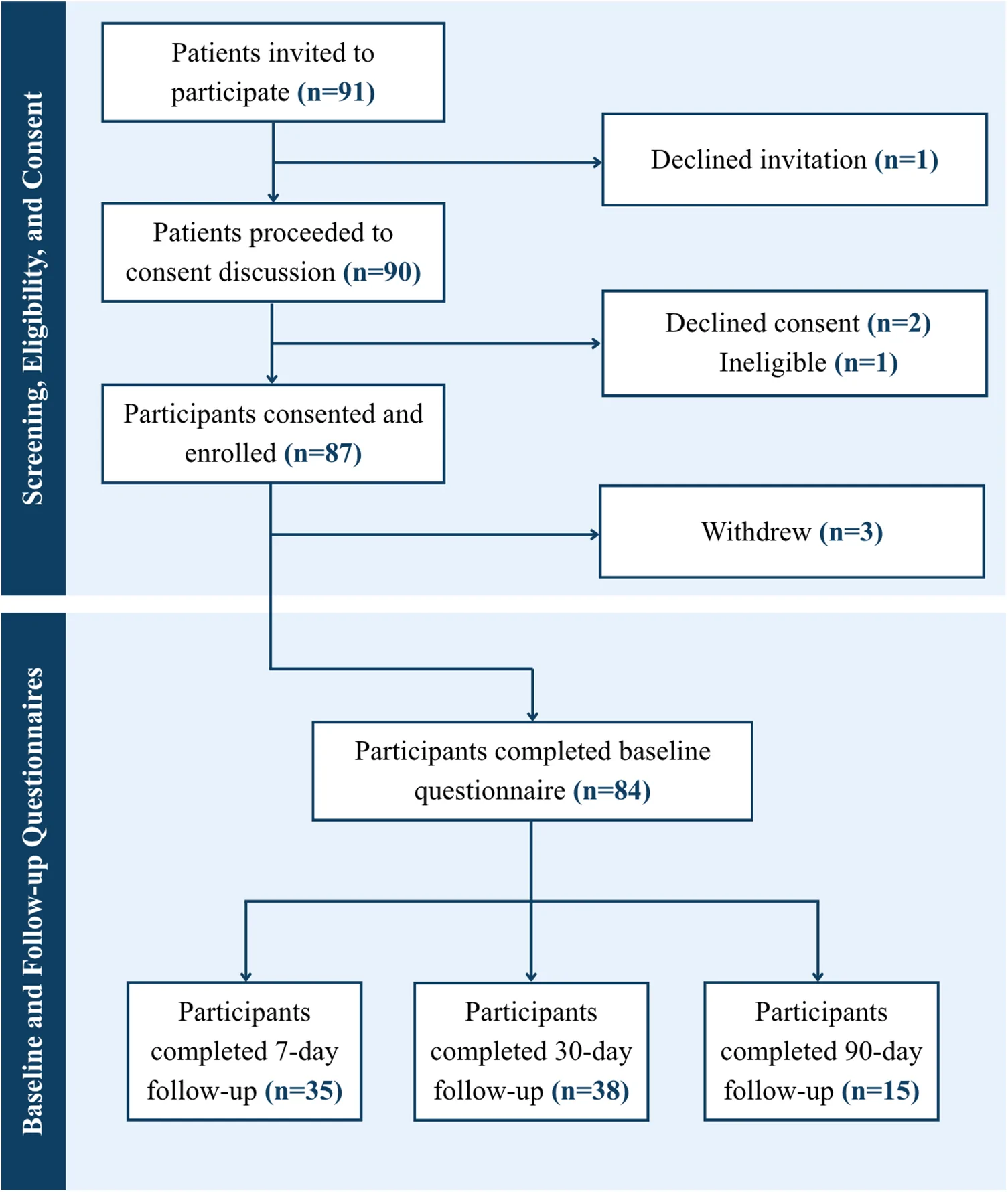
Participant flow through study recruitment, enrolment, and follow-up. Of 91 patients invited to participate, 87 consented and enrolled, and 84 completed the baseline questionnaire. Follow-up completion was assessed separately at each timepoint; follow-up sample sizes represent the number of participants who completed each respective assessment and should not be interpreted as sequential attrition.

**Table 1:** Quantitative results by implementation outcome.

Measure	Results
REACH	
Sociodemographic profile of study participants	Of 84 participants: Indigenous **9.5%**; income below CAD $20,000 **52.4%**; unhoused in the past year **29.8%**; racialised **22.6%**; immigrated to Canada **20.2%**; some dental coverage **64.3%**; had a regular dentist **54.8%**
Study enrolment against unit admissions	87 of 1,226 patients admitted during the recruitment window enrolled in the study (**7.1%)**
Program encounters since December 2022	**777 encounters**
ADOPTION	
Prior awareness of the program	Not at all aware: 39/61 (63.9%)
Knowledge of the program at time of survey	Knew that the program: was run by a registered dental hygienist, **59/61 (96.7%)**; was available two half-days/week, **58/60 (96.7%)**; provided the listed services, **57/61 (93.4%)**; was free to patients, **60/61 (98.4%)**
Referral of patients to the program	Had directed a patient to the program: 54/61 (88.5%)
Integration into unit workflow	Rated the program as well or very well integrated: **54/61 (88.5%)**
ACCEPTABILITY	
Satisfaction with implementation	Satisfied or very satisfied: **57/61 (93.4%)**
Confidence in benefit to patients	Very or extremely confident: **51/61 (83.6%)**
Would recommend the program	Yes: 7 days, **35/35 (100.0%)**; 30 days, **36/37 (97.3%)**; 90 days, **13/15 (86.7%)**
Use of information provided	Yes: 7 days, **31/35 (88.6%)**; 30 days, **31/37 (83.8%)**; 90 days, **13/15 (86.7%)**
APPROPRIATENESS	
Perceived effect on oral health knowledge	Rated moderately to extremely effective: **57/60 (95.0%)**
Perceived effect on routines and motivation	Rated moderately to extremely effective: **55/61 (90.2%)**
Observed change in behaviour or mood	Yes: 43/61 (70.5%)
DETERMINANTS OF THE IMPLEMENTATION PROCESS	
Sustainability and spread	Reported that the program would be valuable in other inpatient units: **57/60 (95.0%)**

Supplementary Table S1 provides complete quantitative results and additional information on data sources, implementation frameworks, and updated Consolidated Framework for Implementation Research determinants. Qualitative findings from the Oral Health Educator interview are reported in the Results section.

### 3.1 Reach

Sociodemographic data collected from study participants indicate that in addition to serving patients on an inpatient mental health unit, the program reached study participants who experience barriers to accessing oral healthcare and who are adversely impacted by determinants of health. Nearly one in ten participants identified as Indigenous (9.5%). Just over half of participants reported an income below CAD $20,000 (52.4%), which is below the 2023 Market Basket Measure (MBM) threshold, Canada's official poverty line, of CAD $26,461 for a one-person household in the Ontario MBM region applicable to Windsor (56). Nearly a third reported being unhoused in the past year (29.8%). Just over one in five reported identifying as racialised (22.6%). Equal proportions of participants reported secondary school and post-secondary education as their highest level of educational attainment (41.7% each). One in five participants identified as having immigrated to Canada (20.2%). Additionally, two-thirds of participants reported having some form of dental coverage (64.3%) and just over half reported having a regular dentist (54.8%).

Of the 1,226 patients admitted during the recruitment window, 87 enrolled in the study. That figure reflects the reach of the study rather than the program's uptake; the OHE was on the unit two half-days each week, so most patients on the unit were never offered the program, and encounters during the recruitment window were not counted separately. The number is also a floor: patients who received the program but did not enrol are not counted, and the program had been delivered on the unit before the study period began. The 777 encounters the OHE recorded since December 2022 (Section 2.3) include repeat encounters, and because they were documented in a free-text template in the hospital electronic medical record (EMR) and not flagged in a queryable field, the precise number of people the program reached is not known. We therefore report these encounters, which reflect uptake of the OHE's services over three years of continuous delivery, despite the OHE being on the unit only two half-days a week, as a proxy for reach. These figures are imperfect, and the reason is worth noting: although the OHE documented every patient encounter, those records cannot be retrieved other than through manual chart abstraction. Retrospective chart abstraction was outside the scope and ethics approval of this study, which covered chart data for consenting participants at baseline only. We return to this in the Discussion.

### 3.2 Adoption

Adoption refers to whether a program is taken up by staff and in the setting in which it is delivered (47, 48). We assessed adoption based on unit staff's perceptions of and engagements with the program, including their awareness of the program, referrals of patients to the program and their perceptions of how well the program was integrated into the existing workflow. We also assessed how the OHE perceived the program's adoption on the unit from their vantage point.

Of the 85 eligible unit staff, 61 completed the survey (71.8%). Before the program began, most respondents (63.9%) had not been aware of it; at the time of the survey, staff knew the program well. Almost all could say who delivered it (96.7% identified the registered dental hygienist), when and where it ran (96.7%), what it offered (93.4%), and that it was free to patients (98.4%) (Access to Knowledge and Information). Most questions offered staff the option of answering that they had not known the program existed; one respondent chose it, on a single question. In all, 88.5% of survey respondents reported having directed a patient to the program, and 88.5% reported that it was well, or very well, integrated into the unit's existing workflow and priorities.

The OHE's account corroborated what staff reported and added to it. Staff support, leadership support, protected time, and supplies were not experienced as barriers to delivering the program. From their perspective, preparation was in place from the outset: “[p]retty much from the beginning, they were ready.” They attributed this to leadership, and to preparation that preceded their arrival, which included a presentation to unit staff about a month before the program began, a site tour with leadership to determine which rooms could be used, and a manager who accompanied them through their first days on the unit. Additionally, the operations manager built a shortcut into the EMR for oral health documentation within the first week. The OHE also reported working collaboratively with nursing staff and social workers, and coordinating with them on whether patients were well enough to be seen. The OHE also reported that patients who had been seen by them referred others, which suggests the program's uptake extended beyond staff referral (Engaging: Innovation Recipients).

### 3.3 Acceptability and appropriateness

Acceptability refers to whether those involved with an innovation, in this case the OHEP, find it agreeable or satisfactory (47). Appropriateness refers to whether the innovation is perceived as a good fit within the delivery setting, for the people receiving it, and for those involved in its delivery, whether directly or indirectly (47). We assessed acceptability from the perspective of unit staff and patients, and appropriateness from the perspective of unit staff and the OHE. We asked staff about their satisfaction with the program and their confidence in its benefit to patients, and we asked patients whether they had used the information provided, and whether they would recommend it to others. We assessed appropriateness drawing on staff's perceptions of the program's effect on patients' oral health knowledge, routines, and motivation, and based on the OHE's account of the program's fit in the setting and for the patients.

Both unit staff and patients found the program acceptable. Staff satisfaction was high: 93.4% were satisfied or very satisfied with how the program was implemented, and 83.6% were very or extremely confident that the program was beneficial in the setting and for patients. Similarly, almost all patients who responded to a follow-up questionnaire said they would recommend the program to others: of those who answered the item, 35 of 35 at 7 days, 36 of 37 at 30 days, and 13 of 15 at 90 days. Most patients also reported using what they had learned: 31 of 35, 31 of 37, and 13 of 15. Nine of the 84 participants responded at all three timepoints; of these, seven said they would recommend the program at every timepoint and five reported using what they had learned at every timepoint.

Both unit staff and the OHE reported finding the program well suited to the unit context and appropriate for the patients. Ninety-five percent of staff perceived the program to be moderately to extremely effective for patients' oral health knowledge, and 90.2% for oral health routines and motivation; 70.5% noticed a change in patients' behaviour or mood, describing patients as seeming “happier,” “brighter,” and “excited.” When asked how oral healthcare fits into a setting that provides mental healthcare, the OHE explained that these were not separate care needs, as many patients were at elevated risk of oral disease and psychiatric medications added to that risk. As they put it, “oral health is linked to systemic health … not treating the mouth as a separate unit, that's where you get better health outcomes” (Mission Alignment). The OHE also recounted that not all patients recognised that they needed oral health support, but that many were thankful for it afterwards.

### 3.4 Feasibility

Feasibility refers to whether an innovation can be carried out in a given setting (47). We assessed feasibility primarily from the OHE's experience of delivering the program, since it was delivered by a single practitioner.

Overall, program delivery was described as uncomplicated. According to the OHE, “as long as you are paying attention to how your patient is responding, and adjusting the session length and the amount of information they are receiving, that is probably the most important piece.” While delivering the sessions themselves posed no major difficulties, two barriers affected program delivery. The first, and the one the OHE identified as the greatest single influence, was reaching patients. Because the OHE was only on the unit two half-days each week, patients admitted and discharged between visits could not be offered the program at all. Relatedly, because patient participation in the program was voluntary, it could proceed only when an eligible patient was both willing and available to take part when the OHE was on the unit. Furthermore, patient availability was itself influenced by the unit's rhythm, since patients in group sessions, meeting clinicians, or with visitors could not be seen. The second barrier was finding space that allowed for patient confidentiality. Support from staff and leadership, protected time, materials, and supplies were not barriers. A new electronic record system took some learning but was manageable with training, and when it was unavailable, delivery continued on paper.

### 3.5 Determinants of the implementation process

In addition to the outcomes and determinants reported above, there were several further determinants that shaped the program's implementation on the unit (49). Two determinants stood out. First, the program was compatible with existing routines on the unit (Compatibility). Both unit staff and the OHE confirmed that the program was integrated into the daily flow of unit work rather than disrupting it. Second, the necessary preparation for the program was in place: unit staff were briefed, the OHE had toured the unit with leadership to determine which rooms could be used, and leadership support was secured before delivery began (Planning; Engaging). A third determinant that facilitated implementation was the design of the innovation itself. The OHEP had been designed to be adaptable and responsive to individual patient needs from the outset and required almost no modification for the inpatient setting (Innovation Adaptability). What's more, because the OHEP was designed for delivery in different settings, delivery continued during a cyberattack that prevented access to the EMR (Innovation Design).

Beyond determinants that shaped program implementation, study data also revealed several factors that speak to the program's potential to be sustained and spread. Unit staff saw benefit in the program and 95.0% of survey respondents shared that they believed that other units would benefit from the program. When asked what would make the program sustainable, staff responded that more frequent OHE presence and wider coverage, so that more patients could be reached and reached earlier in their inpatient stay, would make the program more effective and sustainable. While staff spoke to the value of the program, the OHE spoke to what sustaining and scaling the program would require. When asked whether the program was sustainable, the OHE was unequivocal: with consistent funding it would last. Once again when asked what it would take to spread to other units and populations, the answer was “funding.” Furthermore, the OHE agreed with staff that having someone offering the program more frequently would be a benefit, because a program that only runs two half-days a week would miss patients who arrive on the unit at different times. Thus, ultimately, with dedicated funding, both staff and the OHE felt that the program could be sustained and spread (Available Resources).

## 4 Discussion

### 4.1 Principal findings

The OHEP was implemented successfully on the inpatient mental health unit: staff took it up and it was integrated into their workflow; staff and patients found the program acceptable; staff felt the program was appropriate to the inpatient mental health unit setting; and its delivery in that setting proved feasible. The patients the program reached included people who would not ordinarily receive upstream oral healthcare and who are at higher risk of poorer oral health and overall health outcomes. Staff and the OHE also concurred that the program could be offered more often and reach more patients, though funding was named as the primary barrier. We were unable to assess whether the OHEP changed patients' oral health knowledge, behaviours or quality of life over time, as too few participants completed follow-up questionnaires to support conclusions. Study participant attrition is itself a finding, with implications for how to conduct research with the populations the OHEP was designed to reach.

Several determinants enabled the OHEP's implementation and adoption on the unit, and one constrained the scale at which it could be delivered (49). The program fitted the existing routines of the unit, and both staff and the OHE described it as integrating into the daily flow of unit work rather than disrupting it (Compatibility). Preparation was in place before delivery began: unit staff were briefed, the OHE had toured the unit with leadership to determine which rooms could be used, and leadership support was secured in advance (Planning; Engaging). Staff knew what the program offered, who delivered it, and when it ran, and they referred patients to it (Access to Knowledge and Information). The program had been designed from the outset to respond to the diverse needs and abilities of individual patients, and required almost no modification for the inpatient setting (Innovation Adaptability). The greatest barrier to the program's scale of delivery was its availability. The OHE was on the unit two half-days each week and no amount of fit or preparation could mitigate this (Available Resources).

### 4.2 Integration and its determinants

This study produced information of practical use to others seeking to integrate upstream oral health services into settings where people already receive care. The enabling determinants identified here indicate where efforts to implement integrated oral health programs might be focused: on the preparation that precedes delivery, on the fit between the program and the existing work of the setting, and on ensuring that the staff who are affected by the program know what it offers and who delivers it. Compatibility, in this setting, meant working within the unit's existing rhythm rather than requiring the unit to change it: patients in group sessions, meeting clinicians or with visitors could not be seen at those times, and the OHE coordinated with nursing staff and social workers on whether a patient was well enough. The program's footprint on the unit was correspondingly light: it was delivered by a single registered dental hygienist, present two half-days each week, in whatever room could be found that allowed for patient confidentiality, and the only change to unit systems was a shortcut built into the electronic record for oral health documentation. Staff adopted the program: 88.5% of survey respondents reported having directed a patient to the program, and 88.5% that it was well or very well integrated into the unit's existing workflow and priorities. What our study shows is that the program was integrated into the unit's workflow despite limited funding, which did not bear on whether the program could be implemented or adopted, but did bear on the program's scale of delivery.

Nevertheless, the program reached the people for whom it was designed. All patients on the unit were living with serious mental illness. Among study participants, just over half had an income below CAD $20,000, and nearly a third had been unhoused in the past year. These are the people the literature identifies as being at greatest risk of poorer oral health and poorer overall health outcomes (9, 11, 12). They were able to access this program because it was integrated into a setting in which they already receive care.

### 4.3 Adaptation and fidelity

The OHEP was designed for integration into existing health and community settings, with spread in mind; primary care was its first implementation and the inpatient mental health unit a subsequent one. Assessing whether it remained the same program requires deciding what fidelity means for an innovation of this kind. A program can be judged by whether each of its parts was reproduced exactly, or by whether it remained faithful to its goals and underlying principles. Horton and colleagues suggest that both approaches are legitimate: because complex interventions are social, context-sensitive and dynamic, adaptation by those adopting them should be expected rather than treated as deviation (52). In the case of the OHEP, we argue that fidelity is better understood as faithfulness to the goals and principles of the program (Section 2.3), not component delivery. Consequently, we did not measure fidelity to program components, and could not have: the OHEP specifies a set of core components delivered at an individual patient's pace, with each patient receiving those relevant to their oral health needs, so variation between encounters is a design feature rather than a departure from protocol. That flexibility is not incidental to the populations the program was created to serve. Serious mental illness, dental anxiety, and the fluctuating ability to engage that accompanies acute admission all bear on an individual's capacity and self-efficacy, and the program was designed to accommodate these factors. Asked what mattered most in delivering it, the OHE identified attending to how a patient was responding and adjusting the length of the session and the amount of information accordingly.

Assessed using Horton and colleagues' criteria, i.e., was the program implemented in such a way that it realised its underlying principles and goals, rather than whether each of its components was reproduced in its original form, the OHEP was successfully implemented. The program's delivery on the unit was consistent with each of its three principles. Services were provided at no cost to people on the unit, and more than a quarter of study participants (27.4%) had no dental coverage. Services were integrated into a setting where people at a higher risk of poorer oral health outcomes already receive care: 88.5% of staff reported that the program was well or very well integrated into the unit's workflow, and the OHE worked within the unit's rhythm and alongside unit staff. Services were tailored to the individual: the OHE adjusted each session's length and content to how the patient was responding. The OHEP was designed to anticipate the needs of the individuals and groups it was intended to serve, and in line with the WHO's integrated, people-centred approach to care (37). Given this initial design it required little adaptation for the inpatient setting, and what adaptation was needed did not detract from fidelity to the program's underlying principles and goals. This is a finding in its own right: flexibility and adaptability are not afterthoughts in program design, but precisely the design features that enabled the program to be delivered with fidelity to purpose, which this study bears out. What the OHEP's implementation also demonstrates is that preventive oral healthcare can be designed around the needs of individuals living with serious mental illness.

### 4.4 Strengths and limitations

This evaluation drew on three data sources: patient questionnaires completed by 84 participants, a staff survey completed by 61 of 85 eligible unit staff, and an interview with the person who delivered the program. Because the OHEP was delivered by a single practitioner, that interview represents complete coverage of the delivery workforce rather than a sample of it. Staff and OHE accounts corroborated one another on the determinants that shaped implementation. Each outcome was mapped to its data source and to its corresponding uCFIR determinant. Determinants that shaped implementation without bearing on specific outcomes were recorded alongside them; the construct assignments were independently reviewed by a second author (Supplementary Table S1). The evaluation was conducted in the setting where the program runs, without altering its delivery. Study participants, who are also program recipients, represent populations that are rarely represented in oral health research, and difficult to reach; this difficulty is noted by other studies conducted in inpatient mental health settings (22). Reach was assessed by characterising the sociodemographic profile of study participants and the determinants of health affecting them rather than by enrolment counts alone, which is particularly relevant for a program that aims to advance equity in the delivery of oral healthcare services. Implementation research remains underdeveloped in dentistry and oral healthcare (27) and we found no comparable evaluation of an upstream oral health education program in a comparable setting. This study contributes to addressing the gap in the evidence.

The study has several limitations. It was conducted on one unit at a single hospital, so we cannot say how the program would fare elsewhere. The program was delivered by one educator, and much of what we report about delivery, adaptation, and fit rests on a single account, from someone who co-developed the program and is a co-author of this paper (Section 2.6). The adaptation required for the inpatient setting was minor, so the argument that the program remained faithful to its goals and underlying principles is only mildly tested. Referral was self-reported by staff and was not verified against a record. Staff who responded to the survey (71.8% of those eligible) may have viewed the program more favourably than those who did not. The survey items on acceptability and appropriateness were developed for this study rather than drawn from validated measures, and have not been tested for reliability or validity. Appropriateness was assessed from staff and OHE perspectives only; the follow-up questionnaire was designed to measure change in patients' oral health rather than their view of the program's fit, so patient-reported appropriateness is absent. Patient-reported acceptability items were collected but affected by attrition; nine of the 84 participants responded at every timepoint, and the results are therefore reported separately rather than compared.

We amended the study to an implementation evaluation after the patient data had been analysed (Section 2.1). The patient questionnaires had been designed to measure change in oral health knowledge, behaviours and quality of life, not to assess implementation, which limited what the patient data could tell us about implementation. Had we designed the study as an implementation evaluation at the outset, we would have asked patients different questions about the program and its delivery, including whether it was appropriate to their needs and circumstances. The staff survey and OHE interview were added by amendment in January 2026, after the patient data were available. Staff therefore had to recall, in 2026, how aware they had been of the program before it began in 2022, and these answers may be affected by recall bias. Little else changed in the interval: the program ran continuously, two half-days a week, the mental health department's staff vacancy rate was 1.4% from January 2024 to March 2026 (Windsor Regional Hospital staffing dashboard, unpublished data), and 70.5% of staff respondents had worked on the unit for more than five years. Written and spoken English proficiency was a condition of participation in the study: interpretation was available during a patient's stay on the unit but not for follow-up after discharge, and study documents were not translated. No patient was recorded as excluded on these grounds, and the OHE saw no patients with language barriers on the unit, but we cannot say how many patients on the unit did not speak English. In a study of a program intended to reach people who face barriers to accessing care, the requirement may have excluded a group likely to face them; one in five participants had immigrated to Canada. Study participants may therefore under-represent people who do not speak English, and what we report about who the program reached may not extend to them. Interpretation tools have since become more available and less costly, and a study of this kind should now be able to include people who do not read or speak English.

### 4.5 Implications for research, practice and policy

Our experience raises a question about what kinds of study designs, and what kinds of evidence, are appropriate for programs serving people living with mental illness and adversely impacted by determinants of health, in particular those who also face housing instability, whose contact details change, and whose engagement with the health system is brief and unplanned. The original study design we proposed treated the individual patient as the unit of analysis and required that they remain reachable for three months. On a unit where the average length of stay is approximately 12 days, that was not realistic. The difficulties we encountered are documented elsewhere and in some cases the study design itself already anticipated them and was built to address them. In a Norwegian study of oral health among psychiatric inpatients, recruitment was limited by clinical stability and the capacity to consent (57), and a multi-site study of early psychosis services in Ontario enrolled a third of those it approached and retained the participants it enrolled over twelve months, but did so through a dedicated research infrastructure in programs where participants had ongoing care relationships (58). Many of the conditions that make this population hard to reach in research are also the conditions that make them hard to reach in care: unstable housing, no reliable phone, and fluctuating capacity. Designing a study around the outcome rather than how the populations who are being studied move through services can produce research that cannot answer its own questions. The risk is that evidence stays thinnest where need is greatest, because the designs the field treats as most rigorous require research infrastructures that studies of these populations often do not have.

For those considering delivering an oral health education and promotion program in a comparable setting, both the design of the program and the inner setting context features mattered. The OHEP was built to be adapted to the individual recipient and to its delivery setting, which is why it asked so little of the unit: a room, a way to document, and someone to deliver it. Patients reached the program in three ways: staff referred them, the OHE approached them, and patients who had been seen by the OHE told others about it. Only the first uptake pathway required deliberate preparation. In the OHE's account, implementing the program flexibly, with someone present on the unit who could approach patients directly, enabled uptake to build organically. Staff were briefed before delivery began, leadership was engaged, and the OHE worked within the unit's existing rhythm rather than requiring it to change. The OHE also collaborated with nursing staff and social workers on the unit to know whether a patient could be seen on a given day. Those requirements are modest, and they are the ones we would name as necessary. What preparation and planning could not overcome was program availability: two half-days each week set the ceiling on how many patients could be seen. While we did not ask about program availability directly in any of our data collection, the topic surfaced in both the staff survey responses and the OHE interview. When asked what would improve the program, staff said that patients needed to be seen earlier in the inpatient stay and more often. When asked what most affected program delivery, the OHE said that it was being able to reach patients.

The CDCP (33) is the most significant federal investment in oral healthcare in a generation, and for many Canadians it should close the coverage gap it was designed to close. However, it will not close it for everyone, and in particular for those who are at greatest risk of poorer oral health outcomes. Its conditions, including the annual filing of a tax return and active registration for coverage, must be met before care begins (34), and they are conditions that people experiencing homelessness or housing instability, and those navigating the functional disruptions that accompany serious mental illness, may be least able to meet. Coverage does not by itself guarantee access to care. People who face barriers to accessing oral healthcare can face these barriers whether they have coverage or not (22). A program delivered where people already are does not impose these conditions.

The main barrier to integration, as we noted at the outset, is not evidence but the difficulty of mobilising infrastructure, workforce and funding. As reported above, the resources this program required were minimal. What determined the scale of the program's delivery was how much of the OHE's time was funded, and that funding lever was outside the setting's control. Extending public funding to cover oral health services delivered within health and community-based settings, and not only dental treatment provided to individuals who present for it, should be a policy priority. The WHO has made this case at the level of principle (29); what this study adds is evidence from one inpatient mental health unit that with funding and resources in place, it was feasible, acceptable and appropriate to offer upstream oral healthcare services. A funding model that reaches people only when they present for care does not reach those who are least able to present. The WHO's framework on integrated, people-centred health services asks that care be organised around people rather than around the point at which they seek it (37). For the people this program served, that distinction may bear on whether they receive care at all, and on the oral and overall health outcomes that follow from it.

## 5 Conclusion

This implementation evaluation addresses a gap in the oral health literature, where there is a dearth of evidence about the implementation of integrated oral health programs, in particular the upstream work of education, prevention and promotion. Our findings show that an oral health education and promotion program was integrated into, and delivered in, this inpatient mental health setting, that staff adopted it and almost all patients who responded would recommend it to others, and that it reached people who face barriers to accessing oral healthcare.

Our findings also point to gaps in policy, where coverage-based programs cannot reach people who are unable to meet their conditions of enrolment; in practice, where few programs of this kind exist for this population, and the one we studied relied on funding that sat outside the setting in which it was delivered; and in what counts as evidence, where the study designs the field treats as most rigorous may not account for the lived realities of the people these studies need to recruit. These gaps compound one another. Programs that attempt to align with the WHO-endorsed principles of integrated, people-centred care and the Alma-Ata commitment to essential health services being accessible—delivered where people already are, organised around the person rather than the disease—serve populations that the existing policy and evidence paradigms are not built to reach or evaluate. Consequently, the evidence that would justify more of these programs is hardest to produce, and without it the policy and funding levers that would enable them are unlikely to exist.

## Data Availability

The raw data supporting the conclusions of this article will be made available by the authors, without undue reservation.
