# Supplementary material for: Bringing oral healthcare to where people are: an implementation evaluation of an oral health education program on an inpatient mental health unit
Source: Front Oral Health. 2026 Oct 30;7:1986995. doi: 10.3389/froh.2026.1986995 (PMC13759485; doi:10.3389/froh.2026.1986995)
Supplement: Supplementary file 1 [file Datasheet1.pdf]

# Baseline

Prior to seeing the Oral Health Educator today, how long is it since you last saw a dentist or sought dental care.

- ◯ Less than 6 months
- ◯ 6-12 months
- ◯ More than 1 year but less than 2 years
- ◯ 2 years or more but less than 5 years
- ◯ 5 years or more
- ◯ Never received dental care

What was the reason for your last visit to the dentist?

- ◯ Consultation/advice
- ◯ Pain or trouble with teeth, gums or mouth
- ◯ Treatment/follow-up treatment
- ◯ Routine check-up/treatment
- ◯ Don't know/don't remember

If you didn't visit the dentist, what was the reason?

________________________________________

Do you have any dental coverage?

- ◯ Yes
- ◯ No
- ◯ Used to
- ◯ Do not know/unsure
- ◯ Prefer not to answer

If yes, please specify (optional):

________________________________

How often do you prioritize dental health/oral hygiene?

- ◯ Never
- ◯ Sometimes
- ◯ Often
- ◯ Always
- ◯ Prefer not to answer

How would you rate your dental knowledge?

- ◯ High
- ◯ Medium
- ◯ Low

How would you rate your current oral health?

- ◯ Excellent
- ◯ Very Good
- ◯ Good
- ◯ Fair
- ◯ Poor

**The following three questions are asking you to think back to your knowledge prior to visit with the Oral Health Educator today. There are no right or wrong answers.**

According to you, a regular dental check-up must be:

- ◯ Every 6 months
- ◯ Once a year
- ◯ Only when necessary
- ◯ Do not know
- ◯ Prefer not to answer

Which of the following is your main goal when tooth brushing:

- ◯ Preventing cavities
- ◯ Achievement of cleaner and brighter teeth
- ◯ Removing stains on teeth
- ◯ Removing plaque
- ◯ No goal -- just a component of your hygiene routine
- ◯ Do not know
- ◯ Prefer not to answer

| | |
|---|---|
| The health of one's teeth and mouth affect overall health of body: | ◯ Strongly disagree<br>◯ Disagree<br>◯ Neither disagree nor agree<br>◯ Agree<br>◯ Strongly Agree<br>◯ Don't know<br>◯ Prefer not to answer |

**The following questions are asking you to reflect on your oral health education appointment today. The Oral Health Educator won't be made aware of your individual answers.**

| | |
|---|---|
| Did you learn new information from the oral cancer screening education provided by the dental hygienist? | ◯ Yes<br>◯ No<br>◯ Prefer not to answer |
| You were given educational pamphlets, please comment on their informativeness | ______________________________ |
| Will you be using any of the referrals provided? | ◯ Yes<br>◯ No<br>◯ Maybe<br>◯ Prefer not to answer |
| Will you be using the educational pamphlets provided? | ◯ Yes<br>◯ No<br>◯ Maybe<br>◯ Prefer not to answer |
| What did you learn from the oral health education provided by the dental hygienist? | ______________________________ |
| Would you recommend this program to other people you may know? | ◯ Yes<br>◯ No<br>◯ Prefer not to answer |
| If not, why? | ______________________________ |

**The following questions will be asking about your personal, social information. We're collecting this information to understand who is accessing the Oral Health Program and what needs they might have from the program. We'll also use this information to provide more context to understand your experience and outcomes from the Oral Health Education program. All of the questions are voluntary.**

| | |
|---|---|
| Sex assigned at birth: | ◯ Male<br>◯ Female<br>◯ Other<br>◯ Prefer not to answer |
| If other, please specify: | ________________________ |

| | |
|---|---|
| Gender - How do you identify: | ◯ Man<br>◯ Non-Binary<br>◯ Woman<br>◯ Prefer to self-describe<br>◯ Prefer not to answer |
| Self-describe: | ______________________________ |
| Do you identify as First Nations, Inuk/Inuit and/or Métis? (Select all that apply) | ☐ Yes, First Nations<br>☐ Yes, Inuk/Inuit<br>☐ Yes, Métis<br>☐ No<br>☐ Do not know<br>☐ Prefer not to answer |
| In our society, people are often described by their race or racial background. These are not based in science, but our race may influence the way we are treated by individuals and institutions, and this may affect our health. Which category(ies) best describes you? (Select all that apply): | ☐ Black --African, African Canadian, Afro-Caribbean descent<br>☐ East Asian - Chinese, Japanese, Korean, Taiwanese descent<br>☐ Indigenous - (First Nations, Inuk/Inuit, Métis) ‡ First Nations, Inuk/Inuit, Métis descent<br>☐ Latin American- Hispanic or Latin American descent<br>☐ Middle Eastern - Arab, Persian, West Asian descent (e.g., Afghan, Egyptian, Iranian, Kurdish, Lebanese, Turkish)<br>☐ South Asian - South Asian descent (e.g., Bangladeshi, Indian, Indo-Caribbean, Pakistani, Sri Lankan) Southeast Asian Cambodian, Filipino, Indonesian, Thai, Vietnamese, or other Southeast Asian descent White European descent<br>☐ Another race category - please specify<br>☐ Do not know<br>☐ Prefer not to answer |
| Please specify other race identity: | ______________________________ |
| Have you immigrated from a new country? | ☐ Yes<br>☐ No<br>☐ Prefer not to answer |
| If yes, then when: | ______________________________ |
| Do you currently have health coverage in the country? | ◯ Yes<br>◯ No<br>◯ Prefer not to answer |
| Do you identify with having a disability? | ◯ Yes<br>◯ No<br>◯ Prefer not to answer |
| If yes, what type of disability do you have? (Select all that apply) | ☐ Mobility<br>☐ Emotional/psychological<br>☐ Sensory (hearing, vision)<br>☐ Cognitive<br>☐ Chronic illness<br>☐ Other<br>☐ Prefer not to answer |

| | |
|---|---|
| If other, please specify: | ______________________ |
| Do you have reliable transportation to your dental appointments? | ◯ Yes<br>◯ No<br>◯ Sometimes<br>◯ Prefer not to answer |
| What is your primary form of transportation? | ◯ Non-motorized vehicle (bike, scooter, skateboard)<br>◯ Motorized vehicle (Car, motorcycle, E-bike)<br>◯ Taxi/Cab<br>◯ Public Transit (bus, subway, train)<br>◯ On foot<br>◯ Other<br>◯ Prefer not to answer |
| If other, please specify: | ______________________ |
| Describe your housing arrangement | ◯ I live with Family<br>◯ I live with Partner/Partners<br>◯ I live with Friend/Friends<br>◯ I live with Roommates<br>◯ I reside in a shelter<br>◯ I live alone<br>◯ Other<br>◯ Prefer not to answer |
| If other, please specify: | ______________________ |
| If applicable, please state the first THREE (3) characters of your postal code: (leave blank if prefer not to answer) | ______________________ |
| Have you been to a shelter in the past 12 months for resources? | ◯ Yes<br>◯ No<br>◯ Prefer not to answer |
| If yes, please specify shelter resources used | ______________________ |
| Have you been homeless in the past 12 months? | ◯ Yes<br>◯ No<br>◯ Prefer not to answer |
| If yes, please specify when and for how long: | ______________________ |
| What is your highest completed education level? | ◯ No formal education<br>◯ Primary education (kindergarten to grade 8)<br>◯ Secondary education (grade 9 to grade 12/13)<br>◯ Post-Secondary education (college/university)<br>◯ Other<br>◯ Prefer not to answer |

| | |
|---|---|
| If other, please specify: | ______________________ |
| Are you employed? | ◯ Yes<br>◯ No<br>◯ Prefer not to answer |
| Please specify your employment position (leave blank if prefer not to answer) | ______________________ |
| Which of these categories describes your personal yearly income? | ◯ Less than $10,000<br>◯ Between $10,000 - $20,000<br>◯ Between $20,000-$50,000<br>◯ Between $50,000 - $100,000<br>◯ Between $100,000 - $150,000<br>◯ $150,000+<br>◯ Do not know<br>◯ I prefer not to answer |
| Do you receive income from sources other than your employment? | ◯ Yes<br>◯ No<br>◯ Not applicable<br>◯ I prefer not to answer |
| If yes, please specify: | ______________________ |
| Did you receive social security benefits or disability income in the last year? | ◯ Yes<br>◯ No<br>◯ Not applicable<br>◯ I prefer not to answer |
| Given the information you have provided regarding your social demographics, what types of educational materials or resources would you find most helpful and culturally appropriate in addressing your oral health needs? And do our materials address those needs? (can probe for specific answers based on answers provided to demographic questions ex. For instance, you've identified as having a disability. What types of educational materials or formats would be most helpful for you within your abilities?) | ____________________________ |
| As you know this study requires us to follow up with you in one week and again in one month. Please provide us with the best contact information to reach you, plus any alternate contact information that you authorize us to contact you: | ☐ Phone number<br>☐ Alternate Phone number<br>☐ Do not have access to a phone<br>☐ Email address<br>☐ Alternate Email address<br>☐ Do not have access to an email/computer |
| Phone Number: | ______________________ |
| Alternate Phone Number: | ______________________ |
| Email Address: | ______________________ |

Alternate Email Address: ______________________________
