# Supplementary material for: Bringing oral healthcare to where people are: an implementation evaluation of an oral health education program on an inpatient mental health unit
Source: Front Oral Health. 2026 Oct 30;7:1986995. doi: 10.3389/froh.2026.1986995 (PMC13759485; doi:10.3389/froh.2026.1986995)
Supplement: Supplementary file 2 [file Datasheet2.pdf]

Follow Up Questionnaire

Have you seen a dentist or received dental care since we last spoke?	◯ Yes<br>◯ No<br>◯ Not applicable<br>◯ Prefer not to answer
If yes, did you use the referral provided?	◯ Yes<br>◯ No<br>◯ Not sure<br>◯ Not applicable<br>◯ Prefer not to answer
If yes, please indicate which referral source you utilized, if there were multiple options provided:	________________________________
Did you use the educational pamphlets provided?	◯ Yes<br>◯ No<br>◯ Prefer not to answer
Did you use the information you learned?	◯ Yes<br>◯ I did not use the information<br>◯ I did not learn anything<br>◯ Prefer not to answer
If yes, please specify (optional):	________________________________
Would you recommend this program to other people you may know?	◯ Yes<br>◯ No<br>◯ Prefer not to answer
Please explain why you would not recommend	________________________________
You were given educational pamphlets, please comment on their how informative/helpful they were:	________________________________
Have your medications changed since your last visit with us? *should list for the patient the medications we have documented from their chart<br><br>Auto select N/A if not yet discharged and check chart for meds.	◯ Yes<br>◯ No<br>◯ Not applicable - not yet discharged<br>◯ Prefer not to answer
Please specify (optional):	________________________________
How much do you value dental health /oral hygiene?	◯ Little<br>◯ Neutral<br>◯ A lot<br>◯ Prefer not to answer

How often do you prioritize dental health/ Oral hygiene?	◯ Never<br>◯ Sometimes<br>◯ Often<br>◯ Always<br>◯ Prefer not to answer
According to you, a regular dental check-up must be:	◯ Every 6 months<br>◯ Once a year<br>◯ Only when necessary<br>◯ Do not know<br>◯ Prefer not to answer
Which of the following is your main goal when tooth brushing:	◯ Preventing cavities<br>◯ Cleaner and brighter teeth<br>◯ Removing stains on teeth<br>◯ Removing plaque<br>◯ No goal -- just a component of your hygiene routine<br>◯ Do not know<br>◯ Prefer not to answer
The health of your teeth and mouth affect overall health of body:	◯ Strongly disagree<br>◯ Disagree<br>◯ Neither disagree nor agree<br>◯ Agree<br>◯ Strongly Agree<br>◯ Don't know<br>◯ Prefer not to answer
How often are you currently brushing your teeth?	◯ Once in a day<br>◯ Twice in a day<br>◯ More than twice a day<br>◯ Once a week<br>◯ Twice a week<br>◯ Never<br>◯ Prefer not to answer
How often are you currently flossing between your teeth?	◯ Once in a day<br>◯ Twice in a day<br>◯ More than twice in a day<br>◯ Once a week<br>◯ Twice a week<br>◯ Once a month<br>◯ Never<br>◯ Prefer not to answer
Did you modify the way you brush after the appointment?	◯ Yes<br>◯ No<br>◯ Maybe<br>◯ Prefer not to answer
Since your Oral Health Education appointment, have you observed a reduction in the bleeding of your gums, particularly when brushing your teeth?	◯ Yes, significantly less<br>◯ Yes, somewhat less<br>◯ No change<br>◯ Not sure<br>◯ Not applicable - did not have bleeding<br>◯ Prefer not to answer

Did any of the dry mouth recommendations provided by the oral health educator help manage your dry mouth symptoms?	◯ Yes, the recommendations were highly effective<br>◯ Yes, the recommendations helped somewhat<br>◯ No, the recommendations did not make a noticeable difference<br>◯ I haven't tried the recommendations yet<br>◯ Not sure<br>◯ Not applicable - did not have dry mouth<br>◯ Prefer not to answer
Did you have reduced sensitivity or reduced pain with your teeth or gums since your appointment with the oral health educator?	◯ Yes, significantly reduced sensitivity<br>◯ Yes, somewhat reduced sensitivity<br>◯ No change in sensitivity<br>◯ Not sure<br>◯ Not applicable - did not have sensitivity<br>◯ Prefer not to answer
Did the fluoride varnish or Silver Diamine Fluoride application decrease any of the pain or sensitivity with your teeth.<br>(Only ask if they had the treatment)	◯ Yes, significantly reduced sensitivity<br>◯ Yes, somewhat reduced sensitivity<br>◯ No change in sensitivity<br>◯ Not sure<br>◯ Prefer not to answer<br>◯ Not applicable
Have you performed an oral cancer screening on yourself or actively looked inside your mouth to check the tissues for any changes?	◯ Yes<br>◯ No<br>◯ Not sure<br>◯ Prefer not to answer
Are you now more aware of any changes in your mouth?	◯ Yes<br>◯ No<br>◯ Not sure<br>◯ Prefer not to answer
Did you use any of the resources provided (toothbrush, toothpaste, floss, tongue cleaners, mouth wash etc.) ?<br><br>Auto select N/A if not yet discharged as not given resources until discharge.	◯ Yes<br>◯ No<br>◯ Not applicable - not yet discharged<br>◯ Prefer not to answer
If yes, please specify	__________________________________
How would you rate your dental knowledge?	◯ High<br>◯ Medium<br>◯ Low
How would you rate your current oral health?	◯ Excellent<br>◯ Very Good<br>◯ Good<br>◯ Fair<br>◯ Poor
