# Supplementary material for: Bringing oral healthcare to where people are: an implementation evaluation of an oral health education program on an inpatient mental health unit
Source: Front Oral Health. 2026 Oct 30;7:1986995. doi: 10.3389/froh.2026.1986995 (PMC13759485; doi:10.3389/froh.2026.1986995)
Supplement: Supplementary file 3 [file Datasheet3.pdf]

Oral Health Education Program - Program Implementation Survey (Staff)

CONSENT TO PARTICIPATE IN RESEARCH

Study Title: Evaluation and Impact of an Oral Health Education Program in an Inpatient Setting [WRH REB # 24-481]

- Conflicts of Interest: None to declare

Invitation to Participate:
You are invited to participate in this research survey because you are a staff member of the Mental Health Unit at Windsor Regional Hospital's Ouellette Campus.
This consent form provides you with the information to help you make an informed choice. Please read this document carefully and ask any questions you may have. All your questions should be answered to your satisfaction before you decide whether to participate in this research study. If you would like to, you can talk about this study with other people.
Taking part in this study is voluntary. You do not have to participate at all or you may choose to leave the study at any time.

Purpose of the Study:
The purpose of this survey is to assess staff awareness, understanding, experiences, and barriers related to the Windsor Family Health Team's implementation of the Oral Health Education Program on the mental health unit at Windsor Regional Hospital.

What Participants Will Be Asked to Do:
If you agree to participate in this survey, you will be asked to complete a brief anonymous online survey hosted on REDCap (hosted through the University of Windsor). The survey will take about 5-10 minutes to complete.
All survey questions are optional and your responses will be kept confidential. The survey will be used solely for research and quality improvement purposes.

Potential Risks:
Participating in this study poses minimal risk for participants. While the study team will take precautions to protect your confidentiality, privacy, and security of your data/records, the risk of a privacy breach is never zero. You may skip any question do not wish to answer.

Benefits:
There is no direct benefit from taking part in this study. Feedback will be used to guide research-based discussions and writing, as well as help improve program effectiveness and sustainability.

Costs:
There are no costs associated with participating in this survey

Compensation:
You will not be compensated for participating in this survey.

Confidentiality:
If you decide to participate in this study, the research team will only collect the information they need for this study. Your name, email address, and any other identifying information will not be collected or attached to your survey responses. Your survey answers will remain anonymous to everyone - including your coworkers, the person who shared this link with you, the survey organizers, and research team.
Only authorized members of the research team will have access to your anonymous survey data. No members of the research team or unit, or anyone else, will know if you have participated or what answers you have provided.
Authorized representatives of the following organizations may look at your survey responses at the site where these records are held, to check that the information collected for the study is correct and follows proper laws and ethical guidelines:

- The research ethics board who oversees the ethical conduct of this study in Ontario
- This institution and affiliated sites, to oversee the conduct of research at this location

Survey responses will be submitted collected online in a secure 3rd party web-based application hosted at the University of Windsor called REDCap. Link for REDCap's privacy agreement: https://projectredcap.org/software/mobile-app/privacypolicy/
The results may be published/presented to the scientific community at meetings and in journals. Even though the likelihood that someone may identify you from the study data is very small, it can never be completely eliminated. Data will be retained for 5 years following completion of the study. After this time, it will be destroyed.

Voluntary Participation and Withdrawal:
Participation is completely voluntary. Choosing to participate, or not, will not affect your employment, job conditions, or any aspect of your workplace experience.
You may choose not to participate at all or stop participating (called withdrawal) at any time before submission of the survey. Options for withdrawal include exiting the survey or closing the web browser at any point prior to submitting your responses at the end of the survey. Once your survey has been submitted, your answers cannot be withdrawn because they are anonymous and cannot be linked back to you.

Rights of Research Participants:
You have the right to be informed of the results of this study once the entire study is complete. If you would like to be informed of the results of this study, please let the investigator or research team know. Your rights to privacy are legally protected by federal and provincial laws that require safeguards to ensure that your privacy is respected.

Contact:
If you have any questions about this study, please contact a member of the research team.
If you have questions about your rights as a participant, or about ethical issues related to this study and would like to speak to someone not involved in this study, you may contact the Windsor Regional Hospital Research Ethics Office

Consent Statement
If you have read and understood the information above, and would like to proceed, please press "Next Page"

****

Please select your role on the mental health in-patient unit:	◯ Health Care Provider/Practitioner (Physician, Nurse Practitioner, Nurse)<br>◯ Allied Health Professional (Social Worker, Occupational Therapist)<br>◯ Administration & Management (Manager, Executive, Admin/Clerk)<br>◯ Other<br>◯ Prefer not to answer
Please indicate how many years you have been working in WRH's Ouellette campus mental health unit:	◯ Less than 1 year<br>◯ Between 1-5 years<br>◯ More than 5 years<br>◯ Prefer not to answer
Prior to the implementation, how aware were you that Windsor Family Health Team had an Oral Health Education Program?	◯ Not at all aware<br>◯ Slightly aware<br>◯ Moderately aware<br>◯ Very aware<br>◯ Extremely aware<br>◯ Prefer not to answer
Are you aware that the Oral Health Education Program is run by a registered dental hygienist?	◯ No<br>◯ Yes<br>◯ I did not know this program was offered<br>◯ Prefer not to answer
Are you aware that the Oral Health Education program is available to 3N and 3S one half-day per week each?	◯ No<br>◯ Yes<br>◯ I did not know this program was offered<br>◯ Prefer not to answer

Are you aware that the Oral Health Education Program provides oral/oropharyngeal cancer screening, acute symptom management, education, preventative care, post discharge kits with dental supplies and resources, and referrals?	◯ No<br>◯ Yes<br>◯ I did not know this program was offered<br>◯ Prefer not to answer
If you were NOT aware of the services provided by the Oral Health Education Program, please specify which you were unaware about:	☐ Oral/Oropharyngeal Cancer Screening<br>☐ Symptom Management<br>☐ Education<br>☐ Preventative Care<br>☐ Post-Discharge Kit<br>☐ Referrals<br>☐ All of the above<br>☐ Prefer not to answer
Are you aware that the Oral Health Education Program is free of cost to patients?	◯ No<br>◯ Yes<br>◯ I did not know this program was offered<br>◯ Prefer not to answer
Have you ever directed a patient to the Oral Health Education Program?	◯ No<br>◯ Yes<br>◯ I did not know this program was offered<br>◯ Prefer not to answer
How well do you think the Oral Health Education Program has been integrated with existing clinical workflow and priorities?	◯ Not at all<br>◯ Slightly<br>◯ Moderately<br>◯ Well<br>◯ Very Well<br>◯ I did not know this program was offered<br>◯ Prefer not to answer
If you responded 'Not at all' to integration into clinical workflow and priorities, please specify your concerns:	________________________________
What Oral Health Education Program supports do you think patients on the unit benefit from the most? Select all that apply.	☐ Educational materials (pamphlets/posters)<br>☐ Oral health supplies (toothbrush, toothpaste etc.)<br>☐ Direct interaction with the Oral Health Educator<br>☐ Referrals<br>☐ I did not know this program was offered<br>☐ Prefer not to answer
How effective do you think the Oral Health Program has been in improving patient oral health knowledge?	◯ Not effective<br>◯ Slightly effective<br>◯ Moderately effective<br>◯ Very effective<br>◯ Extremely effective<br>◯ Do not know<br>◯ I did not know this program was offered<br>◯ Prefer not to answer

How effective do you think the Oral Health Education Program has been in improving patient oral health routines or motivation?	◯ Not effective<br>◯ Slightly effective<br>◯ Moderately effective<br>◯ Very effective<br>◯ Extremely effective<br>◯ Do not know<br>◯ I did not know this program was offered<br>◯ Prefer not to answer
Have you noticed any change in patient behaviour or mood after their oral health encounter?	◯ No<br>◯ Yes<br>◯ Do not know<br>◯ I did not know this program was offered<br>◯ Prefer not to answer
If you observed changes in patient behaviour or mood after their oral health encounter, please provide an example:	__________________________________
How satisfied are you with the overall implementation of the Oral Health Education Program in the in-patient mental health unit?	◯ Very dissatisfied<br>◯ Dissatisfied<br>◯ Neither dissatisfied nor satisfied<br>◯ Satisfied<br>◯ Very satisfied<br>◯ I did not know this program was offered<br>◯ Prefer not to answer
From the perspective of your role on the unit, is there anything that could be done to improve the Oral Health Education Program's implementation and sustainability?	____
How confident are you that the Oral Health Education Program is a beneficial offering in the mental health unit?	◯ Not confident<br>◯ Slightly confident<br>◯ Moderately confident<br>◯ Very confident<br>◯ Extremely confident<br>◯ I did not know this program was offered<br>◯ Prefer not to answer
Do you have any additional comments or suggestions for the Oral Health Education Program team?	____
Do you believe the Oral Health Education Program would also be valuable in other inpatient units in the hospital?	◯ No<br>◯ Yes<br>◯ I did not know this program was offered<br>◯ Prefer not to answer
If you believe the Oral Health Education Program would be valuable in other inpatient units, please specify which units:	__________________________________
