# Supplementary material for: Bringing oral healthcare to where people are: an implementation evaluation of an oral health education program on an inpatient mental health unit
Source: Front Oral Health. 2026 Oct 30;7:1986995. doi: 10.3389/froh.2026.1986995 (PMC13759485; doi:10.3389/froh.2026.1986995)
Supplement: Supplementary file 4 [file Datasheet4.pdf]

Supplementary File S4. Oral Health Educator Interview Guide

Introduction:

Thank you for volunteering to participate in an interview to discuss the Oral Health Program. The purpose of the interview is to understand the process, experience, and barriers of the Oral Health Educator, regarding to the Windsor Family Health Team's implementation of the Oral Health Education Program on the mental health unit at Windsor Regional Hospital. Feedback will be used to guide research-based discussions and writing for WRH REB #24-481 "Evaluation and Impact of an Oral Health Education Program in an Inpatient Setting", as well as help improve program effectiveness and sustainability.

Participation is completely voluntary. Choosing to participate, or not, will not affect your employment, job conditions, or any aspect of your workplace experience.

All questions are optional; you may decline answering any questions.

As a reminder, as the sole Oral Health Educator, the information you share will not be anonymous to the research team. In publications, all your direct identifiers will be removed and a pseudonym will be used if any direct reference is made to your statements made in this interview. You will have the option to review and accept or decline any direct quotes proposed to be used in publications.

This interview is also being audio recorded for transcription purposes and audio recording will be deleted once transcription is complete.

Do you agree to move forward with the interview?

Questions:

1. Before we get into the greater discussion, can you please describe your oral health background and how you got involved in the Oral Health Education program?
2. Compared to delivering the program in primary care, how much adaptation was required when implementing the Oral Health Education Program (OHEP) in the inpatient mental health unit (iMHU)?
3. Which of the following areas required adaptation when implementing the OHEP in the iMHU?

☐ Session length or pacing?

☐ Teaching methods or delivery style?

☐ Visual aids/materials?

☐ Communication approach?

☐ Behavioural support strategies?

☐ Safety protocols?

☐ Level of collaboration with other stakeholders?

☐ Follow-up or reinforcement activities?

4. Can you elaborate on why this/these adaptation(s) were needed and what they involved? What do you think were the top 3 most important adaptations?
5. When did the majority of the adaptations occur?

☐ pre-implementation of the primary care model [proactively]

☐ post-implementation of the primary care model [reactively]

6. How clear were the core, non-negotiable components of the OHEP during implementation in the iMHU?
7. How complex did it feel to deliver the OHEP in the iMHU context? Explain your answer.
8. Which patient-related factors influenced your implementation approach?

☐ Literacy?

☐ Cognitive status?

☐ Motivation?

☐ Medication side-effects?

☐ Mood/symptom fluctuation?

☐ Behaviour?

☐ Other?

____

9. What kind of cultural or trauma-informed adaptations did you include in the program?
   a. Probe: If none, were they considered?
   b. Probe: If included, how were they chosen?

10. Rate the extent to which each factor below created barriers during implementation on a scale of 1 (no barrier) to 5 (major barrier):

___ Patient engagement

___ Support from unit staff

___ Support from leadership

___ Scheduling/time

___ Space, materials, equipment

___ EMR or documentation access

___ Safety/environmental constraints

___ Workflow compatibility

   a. Probe: Which barrier had the greatest impact on the implementation of the OHEP in the iMHU and why?

11. Rate the extent to which the following factors positively contributed to the OHEP implementation in the iMHU from 1 (no contribution) to 5 (major positive contribution):

___ Patient engagement

___ Support from unit staff

___ Support from leadership

___ Scheduling/time

___ Space, materials, equipment

___ EMR or documentation access

___ External networks/referral pathways

___ Your professional experience

12. How aligned was OHEP with existing workflows on the iMHU?
   - **a.** Probe: What factors do you think affected the alignment or lack thereof?

13. How would you rate the iMHU's readiness to adopt the OHEP?
   - **a.** Probe: What do you believe was the biggest contributor to the unit's readiness or lack thereof?

14. How prepared did you feel at the implementation start of the OHEP in the iMHU?
   - **a.** Probe: If you felt adequately prepared, what do you feel was the reason(s) for that?
   - **b.** Probe: If you felt unprepared, what do you feel was missing?

15. What new training or knowledge, if any, did you acquire to prepare for the implementation of the OHEP in the iMHU and the population being served?
   - **a.** Probe: If none, do you feel it would have been helpful looking back?
   - **b.** Probe: If yes, do you feel it had an impact on your preparedness?

16. How confident did you feel adapting the OHEP for the iMHU and its patients?

17. How clear was your role on the iMHU, TO YOU, regarding the OHEP delivery?

18. Which stakeholders were engaged in the planning, adaptation, and implementation of the OHEP?

☐ Unit leadership?

☐ Funders?

☐ Academia?

☐ Consultants?

☐ Community support partners?

☐ Patients/previous recipients of the program?

☐ Healthcare providers?

☐ Profession-based associations?

☐ Other? ____

19. How effective was the rollout planning process?
   - **a.** Probe: Were there any incidents that delayed or disrupted the process?

20. Did you have structured opportunities to reflect on what was working and adjust your approach?
   - **a.** Probe: If yes, when/how often did these opportunities arise?
   - **b.** Probe: If yes, did these opportunities result in change?
   - **c.** Probe: If no, do you feel you would have benefited from it?

21. Which aspects of the original primary care OHEP model needed to remain unchanged to maintain fidelity when implementing it in the iMHU?

22. How feasible is the OHEP delivery in the iMHU?

   a. Probe: If infeasible, what factors cause this? Time? Funding? Supplies?

23. Do you think the OHEP is sustainable in the iMHU?

24. What improvements would enhance OHEP implementation in other inpatient or nontraditional settings?

25. What would be required to scale OHEP to other units, facilities, and patient populations?

26. Are there any other points you would like to add about the program or the implementation that we haven't already discussed? Or any other general comments?
