# Supplementary material for: Bringing oral healthcare to where people are: an implementation evaluation of an oral health education program on an inpatient mental health unit
Source: Front Oral Health. 2026 Oct 30;7:1986995. doi: 10.3389/froh.2026.1986995 (PMC13759485; doi:10.3389/froh.2026.1986995)
Supplement: Supplementary file 5 [file Table1.pdf]

Supplementary Table S1. Implementation outcomes, data sources, corresponding uCFIR determinants and results.

Measure	Data source	Implementation outcome	uCFIR determinant	Reported in this study	Results
**REACH**					
Sociodemographic profile of study participants	Patient questionnaire (baseline)	Reach (RE-AIM)	—	Reported descriptively. Describes the characteristics of study participants; representativeness could not be assessed, as comparable data for all patients on the unit were not available.	Of 84 participants remaining in the study: Indigenous 9.5%; income below CAD $20,000 52.4%; unhoused in the past year 29.8%; racialised 22.6%; immigrated to Canada 20.2%; some dental coverage 64.3%; had a regular dentist 54.8%.
Study enrolment against unit admissions	Hospital administrative data; study records	Reach (RE-AIM)	—	Reported as a study-enrolment figure. Not a measure of program reach: the number of patients offered the program during the recruitment window was not recorded.	87 of 1,226 patients admitted during the recruitment window enrolled in the study (7.1%); participant flow Figure 1.
Program encounters since December 2022	Program records (hospital EMR)	Reach (RE-AIM), using uptake of OHE services as a proxy	—	Shows uptake of the OHE's services over three years of continuous delivery since December 2022, despite the OHE being on the unit only two half-days a week. Counts encounters, not unique patients; some patients had more than one encounter and the number of individual patients served is not known.	777 encounters.
**ADOPTION**					
Prior awareness of the program	Staff survey (Q3)	Adoption (RE-AIM)	Access to Knowledge and Information	Reported. Recalled at a single timepoint, so subject to recall bias; most respondents (70.5%) had worked on the unit for more than 5 years (Q2).	Not at all aware: 39/61 (63.9%); moderately: 3/61 (4.9%); very: 4/61 (6.6%); extremely: 9/61 (14.8%); prefer not to answer: 6/61 (9.8%).
Knowledge of the program at time of survey	Staff survey (Q4, Q5, Q6, Q8)	Adoption (RE-AIM)	Access to Knowledge and Information	Reported. Knowledge of the program at the time of the survey (February 2026); not subject to recall bias.	Knew that the program: was run by a registered dental hygienist 59/61 (96.7%); was available two half-days/week, 58/60 (96.7%); provided the listed services, 57/61 (93.4%); was free to patients, 60/61 (98.4%).
Referral of patients to the program	Staff survey (Q9)	Adoption (RE-AIM)	—	Reported. Self-reported; not verified against a referral record.	Yes: 54/61 (88.5%); No: 7/61 (11.5%).
Integration into unit workflow	Staff survey (Q10)	Adoption (RE-AIM)	Compatibility	Reported.	Not at all 0/61 (0.0%); slightly 2/61 (3.3%); moderately 5/61 (8.2%); well 10/61 (16.4%); very well 44/61 (72.1%).
Educator account of adoption on the unit	OHE interview	Adoption (RE-AIM)	Engaging: Innovation Recipients	Reported, including patient-to-patient referral. Single account.	Reported in Results section.
Referral uptake	Follow-up questionnaire	Adoption (RE-AIM)	—	Not reported. Too few responses to report as a proportion.	Not reported.
**ACCEPTABILITY**					

Measure	Data source	Implementation outcome	uCFIR determinant	Reported in this study	Results
Satisfaction with implementation	Staff survey (Q17)	Acceptability (Proctor)	—	Reported.	Very dissatisfied 0/61 (0.0%); dissatisfied 1/61 (1.6%); neither 3/61 (4.9%); satisfied 12/61 (19.7%); very satisfied 45/61 (73.8%).
Confidence in benefit to patients	Staff survey (Q19)	Acceptability (Proctor)	—	Reported.	Not confident 1/61 (1.6%); slightly 2/61 (3.3%); moderately 7/61 (11.5%); very 9/61 (14.8%); extremely 42/61 (68.9%).
Would recommend the program	Follow-up questionnaire	Acceptability (Proctor)	—	Reported per timepoint. Nine participants responded at all three timepoints; results are not compared across timepoints.	Yes, n/N (%): 7 days: 35/35 (100.0%); 30 days: 36/37 (97.3%); 90 days: 13/15 (86.7%).
Use of information provided	Follow-up questionnaire	Acceptability (Proctor)	—	Reported per timepoint; results are not compared across timepoints. Behavioural proxy for acceptability, not a measure of effectiveness.	Yes, n/N (%): 7 days: 31/35 (88.6%); 30 days: 31/37 (83.8%); 90 days: 13/15 (86.7%).
**APPROPRIATENESS**					
Perceived effect on oral health knowledge	Staff survey (Q13)	Appropriateness (Proctor)	—	Staff perception; not a measure of effectiveness.	Not effective 0/60 (0.0%); slightly 2/60 (3.3%); moderately 11/60 (18.3%); very 13/60 (21.7%); extremely 33/60 (55.0%); don't know 1/60 (1.7%).
Perceived effect on routines and motivation	Staff survey (Q14)	Appropriateness (Proctor)	—	Staff perception; not a measure of effectiveness.	Not effective 0/61 (0.0%); slightly 4/61 (6.6%); moderately 16/61 (26.2%); very 6/61 (9.8%); extremely 33/61 (54.1%); don't know 2/61 (3.3%).
Observed change in behaviour or mood	Staff survey (Q15)	Appropriateness (Proctor)	—	Staff observation; not a measure of effectiveness.	Yes 43/61 (70.5%); No 7/61 (11.5%); don't know 11/61 (18.0%).
Fit of the program in the setting and for patients	OHE interview	Appropriateness (Proctor)	Mission Alignment	Reported. Single account.	Reported in the Results section.
**FEASIBILITY**					
Experience of delivering the program	OHE interview	Feasibility (Proctor)	—	Reported. Single deliverer.	Reported in the Results section.
Barriers to delivery	OHE interview	Feasibility (Proctor)	Available Resources	Reported: reaching patients and finding space that allowed for patient confidentiality. Both rated 4 or 5 as barriers.	Reported in the Results section.
Staff support, leadership, protected time, materials, supplies	OHE interview	Feasibility (Proctor)	Available Resources	Reported as not experienced as barriers.	Reported in the Results section.
Electronic record system	OHE interview	Feasibility (Proctor)	Innovation Design	Reported. Delivery continued on paper when the system was unavailable.	Reported in the Results section.
**DETERMINANTS OF THE IMPLEMENTATION PROCESS**					

Measure	Data source	Implementation outcome	uCFIR determinant	Reported in this study	Results
Fidelity to the program's underlying principles and goals	OHE interview; program documentation	Implementation (RE-AIM)	Innovation Adaptability	Assessed against the program's underlying principles and goals rather than the reproduction of its components, following Horton et al. Reported in the Discussion.	Assessed in the Discussion section.
Fit with existing unit routines	Staff survey (Q10); OHE interview	—	Compatibility	Reported. Dual-sourced.	Staff survey: see Integration into unit workflow (Adoption). OHE interview: reported in the Results section.
Preparation before delivery began	OHE interview	—	Planning; Engaging	Reported: staff briefing, site tour with leadership, leadership support secured in advance.	Reported in the Results section.
Adaptation required for the inpatient setting	OHE interview	—	Innovation Adaptability	Reported. Minor adaptation only.	Reported in the Results section.
Sustainability and spread	Staff survey (Q18, Q21); OHE interview	—	Available Resources	Reported with determinants. Maintenance was not assessed in the RE-AIM sense of sustained delivery over time.	Program would be valuable in other inpatient units: Yes: 57/60 (95.0%); No: 1/60 (1.7%); Do not know: 1/60 (1.7%); did not know the program was offered: 1/60 (1.7%) (Q21). Suggestions for sustainability (Q18) and OHE interview: reported in the Results section.
**NOT ASSESSED**					
Change in oral health knowledge, behaviours and quality of life	Patient questionnaire; OHIP-14	Effectiveness (RE-AIM)	—	Not assessed. Too few participants completed follow-up questionnaires to support conclusions (Section 2.1).	Not assessed.
Sustained delivery or institutionalisation over time	—	Maintenance (RE-AIM)	—	Not assessed. Staff and educator perspectives on sustainability are reported with determinants.	Not assessed.
Patient-reported fit to their own needs	—	Appropriateness (Proctor)	—	Not collected. The follow-up questionnaire was designed to measure change in oral health, not perceived fit.	Not collected.
Outer-setting determinants	—	—	—	Not assessed. The evaluation focused on delivery within a single organisation.	Not assessed.
Clinical conditions, diagnoses, medications	Chart abstraction	—	—	Collected to support the original effectiveness design. Not used in the implementation analysis.	Not reported.

OHE, Oral Health Educator; OHIP-14, Oral Health Impact Profile-14; uCFIR, updated Consolidated Framework for Implementation Research. Em dashes indicate that no determinant was assigned or no data source applies. Results are given for measures from the patient questionnaires and staff survey; findings from the interview are reported in the Results section.
